# Curcuminoids Phospholipid Attenuates Osteoarthritis and Protects Cartilage in a Monosodium Iodoacetate-Induced Rat Model

**DOI:** 10.3390/nu18071111

**Published:** 2026-03-30

**Authors:** Hae-Sun Park, Eun-Jung Park, Hae-Jeung Lee

**Affiliations:** 1 Department of Food Science and Biotechnology, Gachon University, Seongnam 13120, Republic of Korea; phs0130@gachon.ac.kr; 2 Institute for Aging and Clinical Nutrition Research, Gachon University, Seongnam 13120, Republic of Korea; 3 Department of Food and Nutrition, Gachon University, Seongnam 13120, Republic of Korea; 4 Department of Health Sciences and Technology, Gachon Advanced Institute for Health Sciences and Technology (GAIHST), Gachon University, Incheon 21999, Republic of Korea; 5 Gachon Biomedical Convergence Institute, Gachon University Gil Medical Center, Incheon 21565, Republic of Korea

**Keywords:** osteoarthritis, curcuminoids phospholipid, cartilage protection, inflammation, NF-κB signaling

## Abstract

**Background/Objectives**: Osteoarthritis is a chronic joint disorder involving the progressive breakdown of articular cartilage, which leads to joint pain and impaired mobility. The present study investigated the effects of curcuminoids phospholipid (CP) on osteoarthritis progression, assessed its cartilage-protective effects, and elucidated the underlying mechanisms. **Methods**: Male Sprague–Dawley rats were randomly allocated to six experimental groups. One group received an intra-articular saline injection as the normal control (NC), while the remaining five groups were injected with monosodium iodoacetate (MIA) and consisted of an MIA control group (MC), a positive control group treated with celecoxib (PC, 3 mg/kg), and three groups treated with CP (31.25, 62.5, or 125 mg/kg). **Results**: Compared with the MC group, CP administration significantly improved pain-related behavior, as assessed by weight-bearing measurements. Micro-computed tomography and histological analyses demonstrated that CP administration mitigated subchondral bone erosion and preserved cartilage integrity. Additionally, the CP treatment significantly reduced markers associated with cartilage degradation, including matrix metalloproteinases and cartilage oligomeric matrix proteins; downregulated the expression of matrix-degrading enzymes; and restored aggrecan expression. Serum levels of inflammatory mediators, including nitric oxide; prostaglandin E_2_; C-reactive protein; and pro-inflammatory cytokines, including interleukin (IL)-6, tumor necrosis factor (TNF)-α, and IL-1β, were reduced following CP administration. Furthermore, CP decreased the activation of nuclear factor kappa B (NF-κB) signaling. **Conclusions**: These findings suggest that CP may be a promising functional agent for osteoarthritis, demonstrating beneficial effects on pain-related outcomes and cartilage integrity, potentially mediated by its anti-inflammatory activity.

## 1. Introduction

Osteoarthritis is one of the most prevalent degenerative joint diseases that significantly impair quality of life and represent a major global cause of disability [1,2]. It is characterized by pain, cartilage degeneration, and inflammation, ultimately leading to functional impairment. The current management of osteoarthritis mainly targets symptom control, commonly using nonsteroidal anti-inflammatory drugs and intra-articular corticosteroid injections [3]. Although effective for temporarily reducing pain, these therapies are associated with systemic and local adverse effects [4,5,6]. Consequently, increasing attention has been directed toward natural compounds that may modulate osteoarthritis pathogenesis while being safe and associated with few side effects [7].

Curcumin has been reported to exert anti-inflammatory and antioxidant effects in various inflammatory diseases [8,9,10]. However, its clinical application is limited by poor bioavailability. Consequently, curcumin formulations with enhanced bioavailability have been developed [11,12]. Curcuminoids phospholipid (CP) is a phytosomal formulation that combines curcuminoids including curcumin, the primary active polyphenolic compound found in turmeric, and phosphatidylcholine. Nevertheless, the mechanisms underlying these effects remain elusive. Furthermore, no study has examined the effects of CP in a rat model of osteoarthritis induced by monosodium iodoacetate (MIA); thus, we explored the mechanisms by which CP exerts its effects in osteoarthritis.

Currently, MIA is the most commonly used chemical in osteoarthritis research, as it acts as a glycolytic inhibitor that blocks glyceraldehyde-3-phosphate dehydrogenase in chondrocytes, leading to cell death [13,14,15]. MIA injection induces osteoarthritis characterized by histological changes and pain-related behaviors within the joint that closely resemble human osteoarthritis [16,17]. Chondrocytes, the primary cellular component of articular cartilage, reduce joint friction and absorb mechanical shock, and support the formation and turnover of the cartilage extracellular matrix (ECM) [18]. As osteoarthritis progresses, cartilage becomes thinner, and chondrocytes are progressively destroyed. Additionally, the synthesis of ECM components, such as collagen and proteoglycans, declines, while matrix degradation mediated by matrix metalloproteinases (MMPs) is accelerated. Furthermore, inflammatory cytokines, including tumor necrosis factor (TNF)-α, interleukin (IL)-6, and IL-1β, promote chondrocyte apoptosis and ECM degradation, and induce the production of additional inflammatory mediators, thereby exacerbating osteoarthritis.

The nuclear factor kappa B (NF-κB) signaling pathway plays a central role in osteoarthritis pathogenesis by regulating inflammatory cytokines and MMPs, thereby promoting cartilage destruction and pain [19]. Activation of this pathway sustains joint inflammation and disease progression. Therefore, targeting the NF-κB signaling pathway represents a promising therapeutic strategy for osteoarthritis. In this study, we aimed to investigate the effects of CP on joint health using an MIA-induced osteoarthritis model and elucidate its mechanism of action, focusing on its effects on NF-κB signaling.

## 2. Materials and Methods

### 2.1. Curcuminoids Phospholipid Preparation

Meriva^®^, a standardized curcuminoids phospholipid (CP) formulation, was obtained from Indena S.p.A. (Milan, Italy). This preparation, comprising a 1:2:2 mixture of curcuminoids (derived from the rhizome of *Curcuma longa* L.), sunflower lecithin, and microcrystalline cellulose, was resuspended in sterile saline prior to oral administration.

### 2.2. Monosodium Iodoacetate-Induced Osteoarthritis Rat Model and Treatment

Sixty Male Sprague–Dawley rats (5 weeks old) were purchased from Orient Bio (Seongnam, Republic of Korea). The rats were maintained in a controlled animal facility at 20–25 °C with 50–55% relative humidity under a 12 h light–dark cycle. Food and water were provided ad libitum. After a 1-week acclimatization period, the rats were randomly assigned to six experimental groups (n = 10 per group): (1) NC, normal control; (2) MC, MIA-injected group; (3) PC, positive control group administered celecoxib 3 mg/kg; (4) CPL, low-dose CP group (31.25 mg/kg); (5) CPM, medium-dose CP group (62.5 mg/kg); and (6) CPH, high-dose CP group (125 mg/kg). The CP administered to each group corresponded to daily intakes of 6.25, 12.5, and 25 mg/kg body weight of highly bioavailable curcuminoids, respectively. To establish an animal model of osteoarthritis, intra-articular injection of MIA (3 mg/50 μL of 0.9% saline) was administered into the right knee one week before initiation of oral administration, whereas the NC group received an equivalent volume of saline. All the groups received oral saline, celecoxib, or CP for 4 weeks, and body weight was measured weekly. At the end of the experiment, the rats were euthanized by CO_2_ inhalation, and blood samples were collected via cardiac puncture. Serum was separated and stored until analysis. Articular cartilage tissues were collected for subsequent experiments. All the procedures were conducted in accordance with the Guidelines for the Care and Use of Laboratory Animals (approval number: GU1-2024-IA0076-00).

### 2.3. Weight-Bearing Distribution Measurements

All the rats were acclimated to an Incapacitance Meter Tester 600 (IITC Life Science, Woodland Hills, CA, USA) for 3 consecutive days prior to weight-bearing distribution measurements in both hind limbs. Measurements were performed on days 0, 7, 14, 28, and 35 in quadruplicate, and the mean values were used for statistical analysis. The weight-bearing ratio was calculated as follows:

$$Weight-bearing\;ratio\;(\%)=\frac{weight\;on\;right\;hind\;limb}{weight\;on\;right\;hind\;limb+weight\;on\;left\;hind\;limb}\times 100$$

### 2.4. Micro-Computed Tomography Analysis

Knee joint tissues were analyzed with a SkyScan 1173 system (Bruker-CT, Kontich, Belgium). Paraffin-fixed samples were scanned at 130 kV and 60 µA using a 1.0 mm aluminum filter. The acquired projection images were reconstructed using the NRecon software (Version 1.7.4.6, Bruker-CT, Kontich, Belgium). The reconstructed datasets were aligned with DataViewer, and quantitative morphometric parameters were subsequently calculated using CTAn software (Version 1.19.4.0, Bruker-CT, Kontich, Belgium) .

### 2.5. Histological Staining and Immunohistochemical Assay

Knee joint tissues were fixed, decalcified, and embedded in paraffin. The paraffin sections were stained with hematoxylin and eosin (H&E) and safranin O–fast green. For immunohistochemistry, the sections were incubated with a primary antibody against collagen type II (Abcam, Cambridge, UK). The stained slides were scanned using a digital slide scanner and analyzed with CaseViewer (ver. 2.1.3, 3DHISTECH, Budapest, Hungary) and Image-Pro Plus (ver. 4.5.0.29, Media Cybernetics Inc., Rockville, MD, USA). Knee osteoarthritis severity was evaluated using the Osteoarthritis Research Society International (OARSI) histopathology scoring system [20]. All the evaluations were performed by a blinded independent pathologist.

### 2.6. Measurement of MMPs, Inflammatory Mediators, and Cytokine Levels in the Serum

Serum biomarkers, including MMP2, MMP3, MMP9, MMP13, collagen type II (Col2), hyaluronic acid (HA), cartilage oligomeric matrix protein (COMP), nitric oxide (NO), prostaglandin E2 (PGE2), C-reactive protein (CRP), and pro-inflammatory cytokines, were measured using commercial ELISA kits (R&D Systems, Minneapolis, MN, USA) following the provided protocol.

### 2.7. RNA Extraction and Quantitative Real-Time PCR

Total RNA was isolated from the cartilage tissues using a previously reported method [21]. The primer sequences used in this study are listed in Table 1.

### 2.8. Western Blot Analysis

Total proteins were isolated from the cartilage tissues, and a Western blot analysis was performed as previously described [21]. Primary antibodies against phosphorylated NF-κB (p-NF-κB), NF-κB, phosphorylated IκB (p-IκB), and glyceraldehyde-3-phosphate dehydrogenase (GAPDH) were obtained from Cell Signaling Technology (Danvers, MA, USA). The band intensities were quantified using the Amersham Imager 680 analysis software (Version 2.0.0, Cytiva, Marlborough, MA, USA) with background subtraction.

### 2.9. Statistical Analysis

All data are presented as mean ± standard deviation (SD). Statistical analyses were performed using GraphPad Prism 10 (GraphPad Software, San Diego, CA, USA). Differences among the groups were analyzed using one-way ANOVA followed by Tukey’s multiple comparisons test. A *p*-value < 0.05 was considered statistically significant.

## 3. Results

### 3.1. CP Improves Weight-Bearing Distribution in MIA-Induced Osteoarthritis Rats

**Figure 1:**
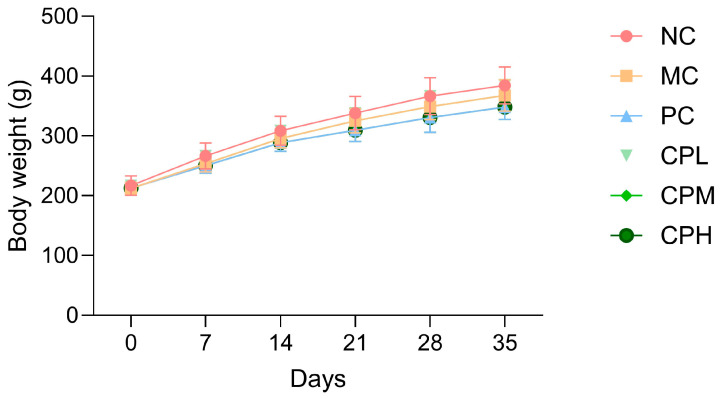
Effect of CP on body weight in MIA-induced osteoarthritis rats. Data are presented as mean ± SD. CP, curcuminoids phospholipid; MIA, monosodium iodoacetate; NC, normal control; MC, MIA-injected group; PC, positive control group administered celecoxib 3 mg/kg and MIA; CPL, low-dose CP group (31.25 mg/kg) with MIA injection; CPM, medium-dose CP group (62.5 mg/kg) with MIA injection; CPH, high-dose CP group (125 mg/kg) with MIA injection.

All the experimental groups showed a gradual increase in body weight over the five-week period, with no statistically significant differences observed in weight gain among the groups. These results indicate that body weight did not influence the study outcomes (Figure 1). Following induction of osteoarthritis by MIA injection, the hindlimb weight-bearing ratio was measured as an indirect indicator of pain. The weight-bearing ratio markedly decreased by day 7 in all the MIA-induced groups compared with the NC group, and this difference persisted for at least 4 weeks. From day 14, both the PC- and CP-administered groups showed an increase in the weight-bearing ratio (Table 2). These findings suggest that CP improves weight-bearing capacity by alleviating osteoarthritis-related pain.

### 3.2. CP Attenuates Morphological Changes in MIA-Induced Osteoarthritis Rats

**Figure 2:**
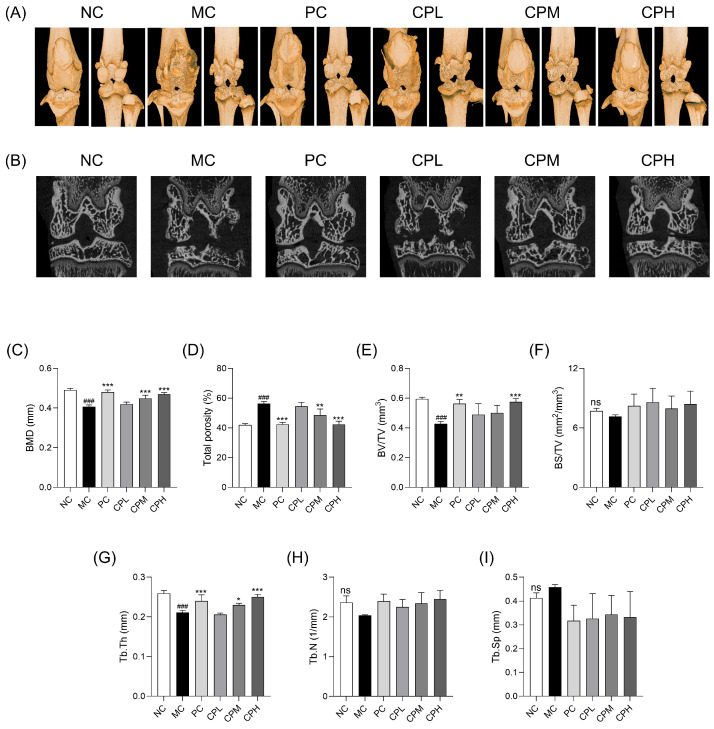
Effect of CP on bone structural damage assessed by micro-CT in MIA-induced osteoarthritis rats:. (**A**) three-dimensional micro-CT images, (**B**) two-dimensional micro-CT images, (**C**) BMD, (**D**) Total porosity, (**E**) BV/TV, (**F**) BS/TV, (**G**) Tb.Th, (**H**) Tb.N, and (**I**) Tb.Sp. Data are presented as mean ± SD. ### *p* < 0.001 vs. the NC group; * *p* < 0.05, ** *p* < 0.01, *** *p* < 0.001 vs. the MC group. ns, not significant; BMD, bone mineral density; BV/TV, bone volume/total volume; CP, curcuminoids phospholipid; MIA, monosodium iodoacetate; CT, computed tomography; Tb.Th, trabecular bone thickness; Tb.N, trabecular bone number; NC, normal control; MC, MIA-injected group; PC, positive control group administered celecoxib 3 mg/kg and MIA; CPL, low-dose CP group (31.25 mg/kg) with MIA injection; CPM, medium-dose CP group (62.5 mg/kg) with MIA injection; CPH, high-dose CP group (125 mg/kg) with MIA injection.

To confirm the morphological structure of the MIA-induced osteoarthritis model, a micro-CT analysis was performed. As shown in Figure 2A,B, the MC group exhibited subchondral bone loss, an irregular articular surface, and trabecular erosion, whereas these MIA-induced changes were markedly alleviated in the PC- and CP-administered groups. Quantitative bone parameters were further analyzed using micro-CT (Figure 2C–I). In the MC group, bone mineral density (BMD), bone volume/total volume (BV/TV), and trabecular bone thickness (Tb.Th) were significantly decreased compared with the NC group, whereas total porosity was increased. In contrast, these parameters were improved in the PC- and CP-administered groups, with the CPH group showing recovery to levels comparable to those of the NC group. However, no significant differences were observed in trabecular number (Tb.N) and trabecular separation (Tb.Sp) among the groups, although Tb.N tended to increase and Tb.Sp tended to decrease in the CP-administered groups compared with the MC group. These results indicate that CP ameliorates morphological changes associated with MIA-induced osteoarthritis.

### 3.3. CP Reduces Histopathological Alterations in MIA-Induced Osteoarthritis Rats

**Figure 3:**
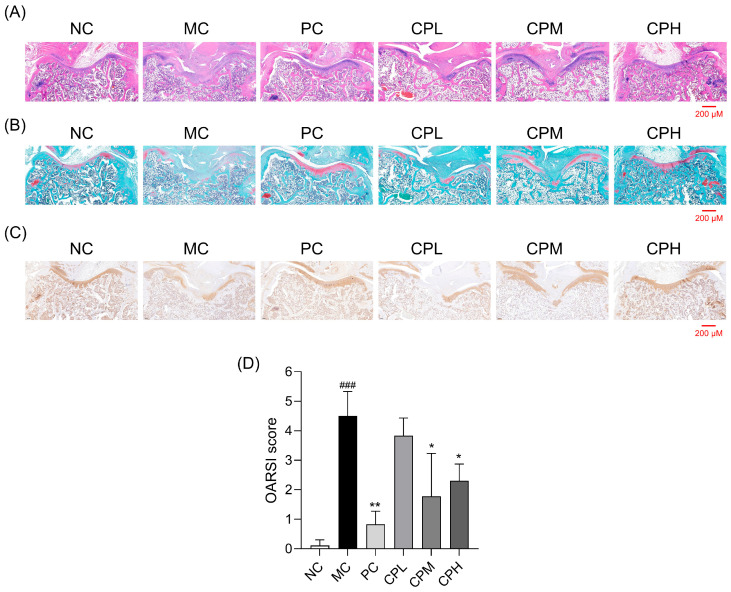
Effect of CP on histological change in MIA-induced osteoarthritis rats: (**A**) hematoxylin and eosin (H&E)-stained sections of rat knee joint (Scale bar = 200 μm); (**B**) safranin O-stained sections; (**C**) immunohistochemical staining for type II collagen; (**D**) quantification of OARSI scores. Data are presented as mean ± SD. ### *p* < 0.001 vs. the NC group; * *p* < 0.05, ** *p* < 0.01 vs. the MC group. CP, curcuminoids phospholipid; MIA, monosodium iodoacetate; NC, normal control; MC, MIA-injected group; PC, positive control group administered celecoxib 3 mg/kg and MIA; CPL, low-dose CP group (31.25 mg/kg) with MIA injection; CPM, medium-dose CP group (62.5 mg/kg) with MIA injection; CPH, high-dose CP group (125 mg/kg) with MIA injection.

To examine histopathological changes in articular cartilage, we performed H&E, safranin-O, and immunohistochemistry staining in the MIA-induced osteoarthritis rats. The cartilage surface in the NC group was smooth and exhibited normal cartilage conditions, whereas the cartilage in the MC group showed pronounced erosion and cartilage damage (Figure 3A). Safranin-O staining revealed a reduced staining area in the MC group, indicating the loss of proteoglycans in the cartilage matrix. The PC- and CP-administered groups showed increased proteoglycan staining area compared with the MC group, suggesting attenuation of cartilage matrix degradation (Figure 3B). Cartilage damage was further assessed by immunohistochemical staining of collagen type II, a major structural protein of cartilage. The MC group exhibited a markedly reduced stained area compared with the NC group, whereas this area was increased in the PC- and CP-administered groups (Figure 3C). OARSI scoring showed that the PC, MEM, and MEH groups had significantly lower scores than the MC group (Figure 3D). These findings indicate that CP alleviates cartilage degeneration in MIA-induced osteoarthritis.

### 3.4. CP Regulates Cartilage Degradation-Related Biomarkers

**Figure 4:**
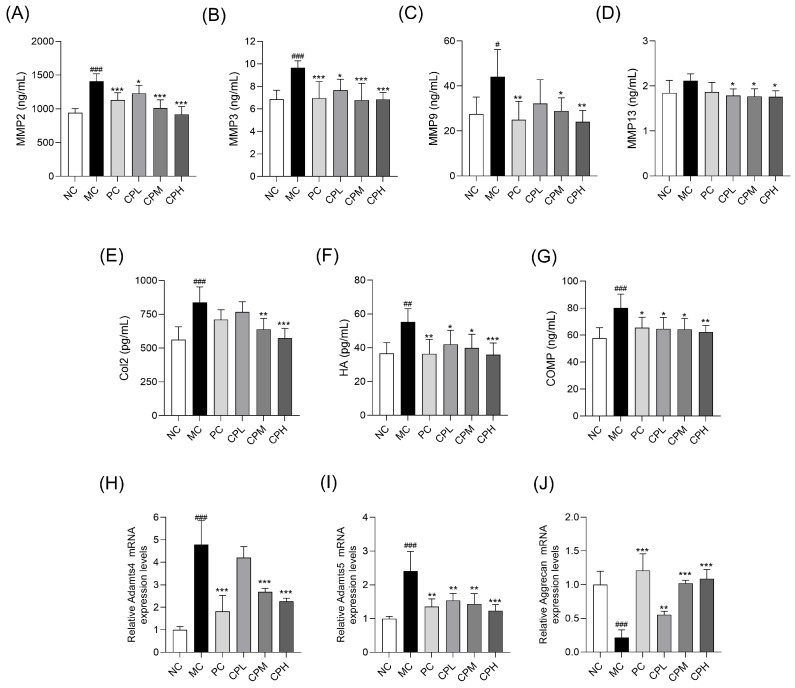
Effect of CP biomarkers of cartilage metabolism in MIA-induced osteoarthritis rats: (**A**–**D**) serum levels of MMP-2, MMP-3, MMP-9, and MMP-13; (**E**) type II collagen; (**F**) HA; (**G**) COMP; and (**H**–**J**) mRNA expression levels of ADAMTS4, ADAMTS5, and aggrecan. Gene expression levels were normalized to GAPDH. Data are presented as mean ± SD. # *p* < 0.05, ## *p* < 0.01, ### *p* < 0.001 vs. the NC group; * *p* < 0.05, ** *p* < 0.01, *** *p* < 0.001 vs. the MC group. CP, curcuminoids phospholipid; MIA, monosodium iodoacetate; NC, normal control; MC, MIA-injected group; PC, positive control group administered celecoxib 3 mg/kg and MIA; CPL, low-dose CP group (31.25 mg/kg) with MIA injection; CPM, medium-dose CP group (62.5 mg/kg) with MIA injection; CPH, high-dose CP group (125 mg/kg) with MIA injection; MMP, matrix metalloproteinase; HA, hyaluronic acid; COMP, cartilage oligomeric matrix protein; Adamts, a disintegrin and metalloproteinase with thrombospondin motifs.

To evaluate cartilage degradation and joint metabolism in MIA-induced osteoarthritis, serum biomarkers associated with cartilage turnover were measured. As shown in Figure 4A–D, the serum levels of MMP-2, MMP-3, MMP-9, and MMP-13 were significantly increased in the MC group, whereas these levels were significantly lower in the CP-administered groups than in the MC group. In addition, the serum levels of Col2, HA, and COMP were significantly increased in the MC group and markedly decreased in the CP-administered group (Figure 4E–G). Furthermore, mRNA expression of a disintegrin and metalloproteinase with thrombospondin motifs 4 and 5 (ADAMTS4 and ADAMTS5), the major aggrecanases, was significantly elevated in the MC group, while aggrecan mRNA expression was markedly decreased. In contrast, the CP treatment significantly suppressed the expression of Adamts4 and Adamts5 and restored aggrecan expression compared with the MC group (Figure 4H–J). These results indicate that CP exerts chondroprotective effects by inhibiting matrix degradation and regulating ECM metabolism.

### 3.5. CP Suppresses Inflammatory Mediators in the Serum of MIA-Induced Osteoarthritis Rats

**Figure 5:**
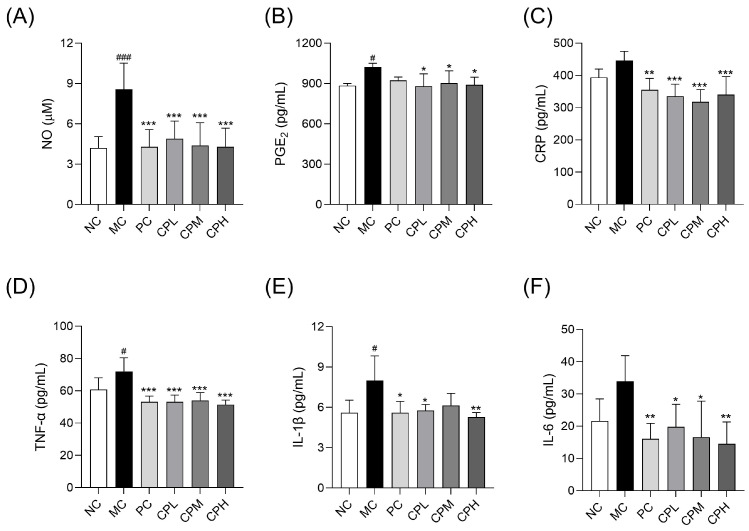
Effect of CP on inflammatory factors in MIA-induced osteoarthritis rats: (**A**) NO levels. (**B**–**F**) The serum levels of inflammatory biomarkers (PGE_2_, CRP, TNF-α, IL-1β, and IL-6). Data are presented as mean ± SD. # *p* < 0.05, ### *p* < 0.001 vs. the NC group; * *p* < 0.05, ** *p* < 0.01, *** *p* < 0.001 vs. the MC group. CP, curcuminoids phospholipid; MIA, monosodium iodoacetate; NC, normal control; MC, MIA-injected group; PC, positive control group administered celecoxib 3 mg/kg and MIA; CPL, low-dose CP group (31.25 mg/kg) with MIA injection; CPM, medium-dose CP group (62.5 mg/kg) with MIA injection; CPH, high-dose CP group (125 mg/kg) with MIA injection; NO, nitric oxide; PGE_2_, prostaglandin E_2_; CRP, C-reactive protein; TNF-α, tumor necrosis factor-alpha; IL, interleukin.

Inflammation plays a crucial role in the onset and progression of osteoarthritis [22]. Accordingly, serum levels of NO, PGE_2_, CRP, TNF-α, IL-1β, and IL-6 were evaluated (Figure 5). All the inflammatory mediator biomarkers were significantly attenuated by the CP treatment compared with the MC group. Collectively, these results suggest that CP inhibited osteoarthritis progression by suppressing inflammatory mediator production.

### 3.6. CP Inhibits NF-κB Activation in MIA-Induced Osteoarthritis Rats

**Figure 6:**
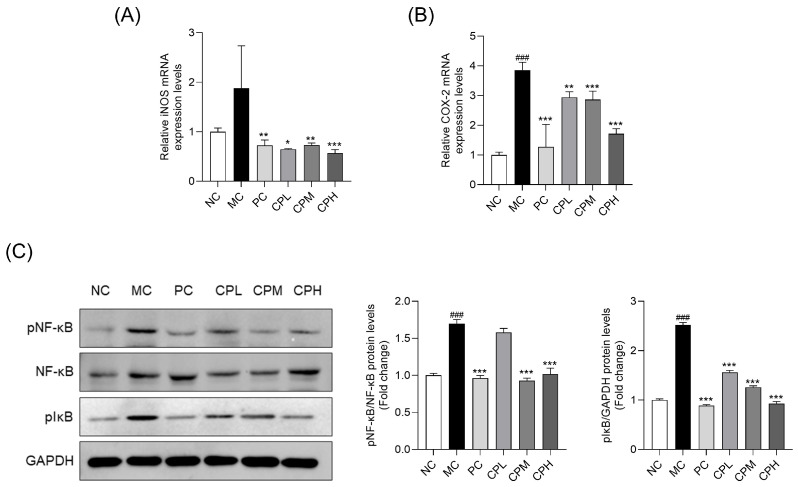
Effect of CP on inflammatory responses in MIA-induced osteoarthritis rats: (**A**,**B**) mRNA expression levels of iNOS and COX-2. (**C**) Western blot analysis of protein expression and phosphorylation levels for NF-κB and IκBα. Relative protein expression levels are presented as fold changes compared to the NC group. Data are presented as mean ± SD. ### *p* < 0.001 vs. the NC group; * *p* < 0.05, ** *p* < 0.01, *** *p* < 0.001 vs. the MC group. CP, curcuminoids phospholipid; MIA, monosodium iodoacetate; NC, normal control; MC, MIA-injected group; PC, positive control group administered celecoxib 3 mg/kg and MIA; CPL, low-dose CP group (31.25 mg/kg) with MIA injection; CPM, medium-dose CP group (62.5 mg/kg) with MIA injection; CPH, high-dose CP group (125 mg/kg) with MIA injection; NF-κB, nuclear factor kappa-light-chain-enhancer of activated B cells; pIκBα, phospho-inhibitor of kappa B alpha.

To evaluate inflammatory responses in MIA-induced osteoarthritis, we examined the expression of inflammatory mediators and the activation of the NF-κB pathway. The mRNA expression levels of inducible nitric oxide synthase (iNOS) and cyclooxygenase-2 (COX-2) were significantly reduced in the CP-administered groups compared with the MC group. (Figure 6A,B). Protein expression levels of p-NF-κB and p-IκBα were significantly elevated in the MC group compared with the NC group. Specifically, the levels of p-NF-κB and p-IκBα increased by approximately 1.70- and 2.51-fold, respectively, indicating increased NF-κB-related inflammatory activity in MIA-induced osteoarthritis. However, expression levels of these proteins were significantly reduced in the CP-administered groups. These findings suggest that CP may be associated with modulation of NF-κB-related inflammatory signaling (Figure 6C).

## 4. Discussion

Osteoarthritis is a chronic inflammatory disorder characterized by cartilage and bone degeneration [23]. Although its clinical manifestations are well recognized, the mechanisms underlying the suppression of structural cartilage damage remain insufficiently understood. Previous clinical studies with Meriva^®^ have reported beneficial effects in patients with osteoarthritis. In a 3-month study, Belcaro et al. reported that Meriva^®^ supplementation was associated with improvements in clinical outcomes, including reduction in global Western Ontario and McMaster Universities Arthritis Index (WOMAC) scores and CRP levels in participants with elevated inflammatory status [24]. In a longer-term study, similar improvements in symptoms and function were observed during an 8-month period, supporting the potential role of Meriva^®^ as a complementary treatment for osteoarthritis management [25]. However, curcuminoids formulations may show limited systemic bioavailability, and clinical outcomes may vary across studies. Therefore, further studies are required to clarify the clinical relevance of these findings. In the present study, we provide mechanistic evidence that CP suppresses NF-κB signaling in an MIA-induced osteoarthritis model, suggesting a potential anti-inflammatory mechanism underlying its effects.

Although the MIA-induced osteoarthritis model does not fully recapitulate human osteoarthritis, it is widely used as a preclinical model to investigate histological and pathophysiological changes in articular cartilage [26,27]. Disease progression in the MIA model is much faster than in human osteoarthritis, as it is primarily driven by rapid chondrocyte death. Therefore, understanding the similarities and differences between the MIA model and human osteoarthritis, together with careful selection of experimental time points according to therapeutic targets, is important for improving the clinical relevance of evaluating potential candidates [28,29]. In this study, the micro-CT analysis demonstrated that CP administration markedly attenuated cartilage damage and bone loss in the MIA-induced osteoarthritis rats, suggesting a protective effect of CP on joint structural integrity. Proteoglycans and type II collagen are essential for preserving the mechanical integrity and functional properties of articular cartilage, which is composed of the major ECM components [30,31]. In this study, we confirmed that MIA injection administration reduced proteoglycan and type II collagen levels, whereas CP administration attenuated these effects. This pattern is consistent with previous reports on MIA-induced osteoarthritis models [32,33]. Collectively, these findings indicate that CP can inhibit ECM degradation and exert a cartilage-protective effect.

Recent studies have emphasized the critical role of inflammation in osteoarthritis progression, reporting elevated levels of inflammatory mediators and cytokines in the blood, synovial fluid, and cartilage of patients [34]. Our results demonstrated that MIA injection significantly increased the levels of inflammatory cytokines and mediators, including CRP, PGE_2_, and NO. In particular, IL-1β, a key cytokine in osteoarthritis, promotes the expression of MMPs and induces the degradation of ECM proteins, including type II collagen and aggrecan [35,36]. Additionally, TNF-α promotes ECM degradation in chondrocytes [37]. In addition, synovial inflammation is recognized as an important component of osteoarthritis pathophysiology. Inflammatory mediators released from synovial tissues can further exacerbate catabolic responses within the joint, thereby promoting cartilage degradation [38]. Therefore, evaluating synovial inflammation may provide a more comprehensive understanding of osteoarthritis progression. Collectively, these inflammatory mediators accelerate cartilage degeneration and promote ECM degradation by upregulating MMP expression, ultimately leading to articular cartilage destruction [39,40]. MMPs directly mediate ECM degradation in osteoarthritis and are responsible for structural cartilage damage. Specifically, MMP-2 and MMP-9 degrade denatured collagens, MMP-3 breaks down various ECM components and activates other MMPs, and MMP-13 serves as the major collagenase in the cartilage [41,42]. In the present study, levels of MMP-2, MMP-3, MMP-9, and MMP-13 were elevated in the MC group, whereas they were reduced in the CP-administered groups. These findings suggest that CP exerts its protective effects by inhibiting inflammatory cytokines and inhibiting ECM degradation.

NF-κB is a key transcription factor that regulates the expression of numerous inflammatory genes, and suppression of this pathway may contribute to attenuating the progression of osteoarthritis [43]. A previous study using an MIA-induced osteoarthritis model demonstrated that inhibition of the NF-κB signaling pathway reduced inflammatory responses and joint pathology [44], highlighting the critical role of this pathway in modulating osteoarthritis-related inflammation. Curcumin has been reported to exert anti-inflammatory effects in various inflammatory diseases through modulation of the NF-κB signaling pathway, particularly by inhibiting the toll-like receptor 4 (TLR4)/NF-κB signaling cascade [45,46,47]. In the present study, CP significantly modulated several downstream inflammatory mediators that are commonly regulated by NF-κB signaling. These findings suggest that the anti-osteoarthritic effects of CP may be associated with suppression of NF-κB-mediated inflammatory signaling. Although upstream signaling pathways were not directly investigated in this study, the observed modulation of NF-κB-related inflammatory responses suggests that upstream pattern recognition receptors, such as TLR4, might be involved, as reported in similar contexts [48]. Activation of the NF-κB pathway enhances the expression of inflammatory cytokines and induces the expression of iNOS and COX-2, thereby increasing the production of NO and PGE_2_ [49,50]. Furthermore, activation of the NF-κB pathway promotes MMPs and aggrecanases, such as Adamts4 and Adamts5, which suppress ECM synthesis and accelerate cartilage matrix degradation [51,52]. In the present study, CP significantly reduced the expression of inflammatory mediators, including iNOS and COX-2, as well as catabolic factors associated with cartilage degradation. These findings suggest that CP may attenuate inflammatory and catabolic responses associated with NF-κB-related inflammatory signaling.

## 5. Conclusions

In this study, CP alleviated pain-related behaviors and preserved cartilage integrity in rats with MIA-induced osteoarthritis. The CP treatment was also associated with reduced inflammatory responses and decreased expression of cartilage-degrading factors. These findings suggest that CP may exert anti-inflammatory and chondroprotective effects in osteoarthritis.

## Figures and Tables

**Table 1:** Primer sequences used for quantitative PCR.

Gene	Primer Sequences (5′-3′)	Accession Number
Adamts4	F: GCCAGCAACCGAGGTCCCAT	NM_023959.1
	R: TTGGCAGCGGCGGCCATGAC	
Adamts5	F: CACGACCCTCAAGAACTTTTGC	NM_198761.2
	R: TCACATGAATGATGCCCACATAA	
Acan	F: CAGAAACCTATGATGTCTAC	NM_022190.2
	R: CAGCCAGCATAGCACTTGTC	
iNOS	F: CACCACCCTCCTTGTTCAAC	NM_001429940.1
	R: CAATCCACAACTCGCTCCAA	
COX-2	F: CCAGCAGGCTCATACTGATAGGA	NM_017232.4
	R: GCAGGTCTGGGTCGAACTTG	
GAPDH	F: GTGGACCTCATGGCCTACAT	NM_017008.4
	R: TGTGAGGGAGATGCTCAGTG	

Adamts, a disintegrin and metalloproteinase with thrombospondin motifs; Acan, Aggrecan; iNOS, inducible nitric oxide synthase; COX-2, cyclooxygenase-2; GAPDH, Glyceraldehyde-3-phosphate dehydrogenase.

**Table 2:** Effect of CP on the changes in hind limb weight-bearing in MIA-induced osteoarthritis rats.

Group						
Days	NC	MC	PC	CPL	CPM	CPH
0	50.13 ± 0.93 ^ns^	49.98 ± 1.96	50.14 ± 1.94	49.90 ± 1.15	50.75 ± 1.03	49.36 ± 1.38
7	50.09 ± 0.97	33.59 ± 1.47 ^###^	34.86 ± 2.54	34.65 ± 3.67	34.17 ± 2.96	34.46 ± 3.72
14	49.14 ± 1.52	31.73 ± 5.74 ^###^	38.48 ± 1.92 ***	34.99 ± 2.19	36.46 ± 2.93 *	37.70 ± 1.57 ***
21	50.09 ± 0.98	32.76 ± 2.63 ^###^	38.41 ± 1.60 ***	36.24 ± 1.59 **	38.46 ± 2.50 ***	38.96 ± 1.55 ***
28	49.98 ± 1.00	32.33 ± 2.79 ^###^	39.27 ± 3.12 ***	36.05 ± 2.35	38.65 ± 3.60 **	39.22 ± 6.12 ***
35	49.59 ± 1.47	32.78 ± 1.94 ^###^	41.17 ± 4.97 **	36.19 ± 3.12	39.42 ± 4.72 *	41.38 ± 5.22 **

Data are presented as mean ± SD. ^###^ *p* < 0.001 vs. the NC group; * *p* < 0.05, ** *p* < 0.01, *** *p* < 0.001 vs. the MC group. ns, not significant; CP, curcuminoids phospholipid; MIA, monosodium iodoacetate; NC, normal control; MC, MIA-injected group; PC, positive control group administered celecoxib 3 mg/kg and MIA; CPL, low-dose CP group (31.25 mg/kg) with MIA injection; CPM, medium-dose CP group (62.5 mg/kg) with MIA injection; CPH, high-dose CP group (125 mg/kg) with MIA injection.

## Data Availability

Data will be made available upon request.

## Abbreviations

ADAMTS	A disintegrin and metalloproteinase with thrombospondin motifs
BMD	Bone mineral density
BV/TV	Bone volume/total volume
COMP	Cartilage oligomeric matrix protein
COX-2	Cyclooxygenase-2
CP	Curcuminoids phospholipid
CRP	C-reactive protein
CT	Computed tomography
HA	Hyaluronic acid
IL	Interleukin
iNOS	Inducible nitric oxide synthase
MC	MIA-injected group
MIA	Monosodium iodoacetate
MMP	Matrix metalloproteinase
NC	Normal control
NF-κB	Nuclear factor kappa-light-chain-enhancer of activated B cells
NO	Nitric oxide
PC	Positive control group
PGE_2_	Prostaglandin E_2_
IκBα	Inhibitor of kappa B alpha
Tb.N	Trabecular bone number
Tb.Th	Trabecular bone thickness
TLR	Toll like receptor
TNF-α	Tumor necrosis factor-alpha
